# SAPHO Syndrome Masquerading as Metastatic Breast Cancer

**DOI:** 10.1155/2018/3168452

**Published:** 2018-03-20

**Authors:** Devin Malik, Richa Handa, Andrew Petraszko, Sheela Tejwani

**Affiliations:** ^1^Department of Hematology Oncology, Henry Ford Hospital, 2799 W. Grand Blvd., Detroit, MI 48202, USA; ^2^Department of Internal Medicine, Henry Ford Allegiance Health, 205 N. East Ave., Jackson, MI 49201, USA; ^3^Department of Radiology, Henry Ford Hospital, 2799 W. Grand Blvd., Detroit, MI 48202, USA

## Abstract

SAPHO syndrome is a rare clinical entity composed of synovitis, acne, pustulosis, hyperostosis, and osteitis (SAPHO). We describe a case of SAPHO syndrome masquerading as metastatic breast cancer in a patient with localized breast cancer who presented with cord compression. There was no pathologic evidence of metastatic cancer; however, a bone scan indicated osseous involvement. After multidisciplinary review of images and with additional findings of pustulosis and acne, a clinical diagnosis of SAPHO was made.

## 1. Introduction

SAPHO syndrome is a constellation of multiple symptoms and signs including synovitis, acne, pustulosis, hyperostosis, and osteitis. It was first described in 1961 by Windam et al. [1], and the acronym was first coined in 1987 by Chamot et al. [2]. It has different names including nonbacterial osteitis, sternoclavicular hyperostosis, pustulotic arthro-osteitis, spondyloarthritis hyperostotica pustulopsoriatica, and chronic recurrent multifocal osteomyelitis [3, 4]. The annual prevalence of SAPHO syndrome is estimated at 1/10,000 in Caucasians [5]. It is a rare clinical entity, and a high index of suspicion is required to diagnose this disease. We present a case in which a patient with invasive breast cancer had a collection of findings including joint pain, acne, and rash involving palms and soles with imaging showing osseous lesions at multiple sites and diagnosed with SAPHO syndrome.

## 2. Case

**Figure 1:**
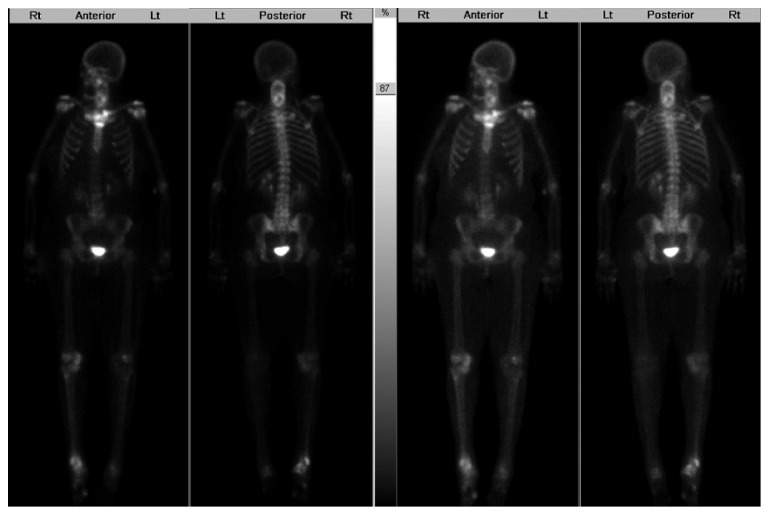
Anterior and posterior planar images of a ^99m^Tc MDP bone scan show increased radiotracer uptake within the medial left clavicle and manubrium centered upon the left sternoclavicular joint and bilateral 1st costomanubrial joints. There is also increased radiotracer uptake with the cervical spine.

**Figure 2:**
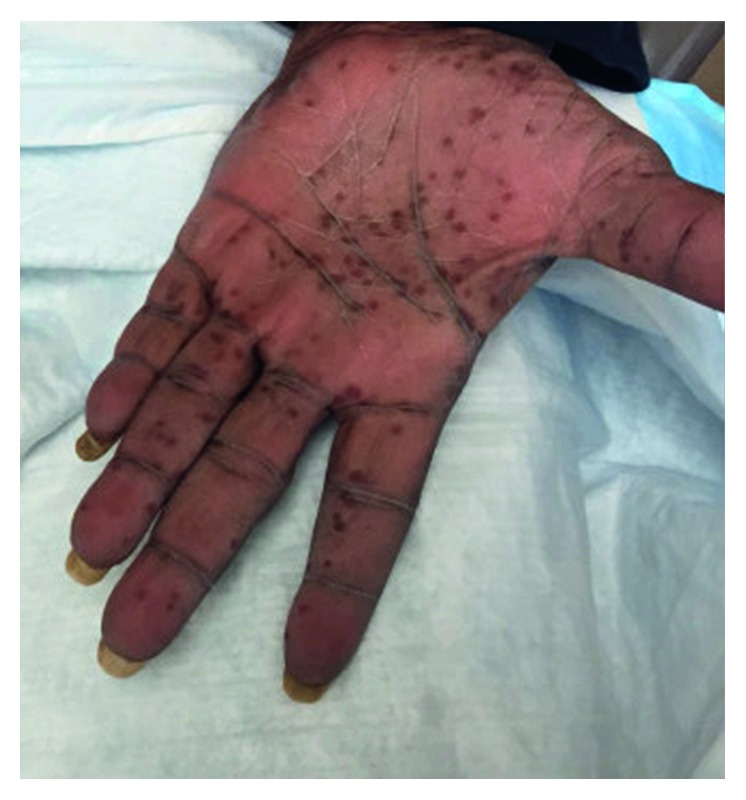
Photograph of the left palm demonstrating healed pustules. Similar findings were on the right palm.

**Figure 3:**
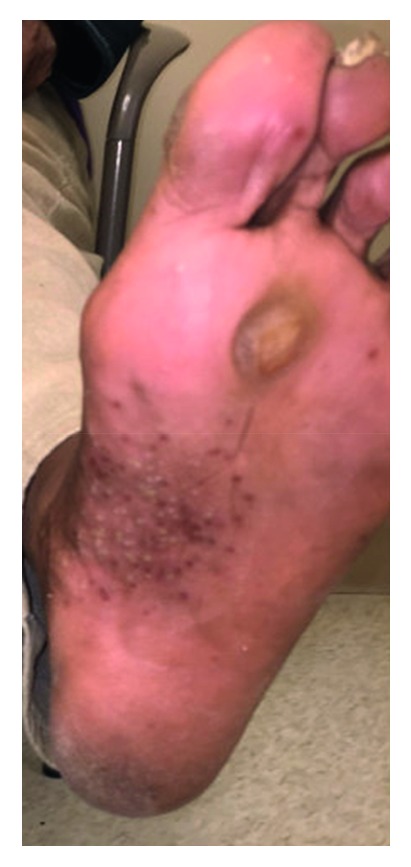
Photograph of the left sole lesions demonstrating pustulosis. Similar findings were on the right sole.

A 59-year-old African American lady with a history of newly diagnosed invasive ductal carcinoma of the breast, stage 1A (pT1b, N0, M0), estrogen receptor and progesterone receptor positive, and Her-2 negative status post lumpectomy and radiotherapy presented to the hospital with complaints of multiple falls and weakness in both arms and legs and incontinence of urine. Complete blood count revealed a neutrophilic leukocytosis of 16.6K/*μ*L (3.8–10.6K/*μ*L), hemoglobin of 13 g/dL (12.0–15.0 g/dL), and platelet of 357K/*μ*L (150–140K/*μ*L). Complete metabolic profile was normal. The patient underwent magnetic resonance imaging which showed osseous lesions involving the cervical spine, thoracic spine, and lumbar spine. Sclerotic lesions were also observed within the left clavicle, sternum, and left T12 proximal rib. The patient underwent X-rays of axial and appendicular skeleton revealing sclerotic appearance of anterior L4 and L5. There was also abnormal appearance of the distal left clavicle, medial right, and left clavicles as well as the sternum. There was no evidence of periostitis. Because of cord compression, the patient underwent vertebral laminectomies which came back negative for metastatic disease and negative for bacteria. The patient also had a bone scan that showed increased radiotracer uptake within the medial left clavicle and manubrium centered upon the left sternoclavicular joint and bilateral 1st costomanubrial joints (Figure 1). There was also increased radiotracer uptake with the cervical spine. The patient also gave a history of rash involving palms and soles (Figures 2 and 3) and facial acne and was seen by a dermatologist in the past and diagnosed with palmoplantar pustular dermatosis. Cultures of these pustules were negative. In light of negative biopsies from surgical specimens and given low-stage breast cancer, an alternative diagnosis for her bone lesions was sought. A comprehensive joint exam was done to evaluate for any bony abnormalities. There was full range of motion of spine, shoulders, elbows, wrists, fingers, hips, knees, and ankles. There was no active swelling, tenderness, soft tissue nodules, or synovitis at any joint. There was tenderness to palpation over the sternum and sternoclavicular joints. There was no malar rash or mouth ulcers, or alopecia.

Plasma cell dyscrasia was ruled out by serum and urine protein electrophoresis and a normal bone marrow biopsy and skeletal radiographic survey. Given her joint pain, bone scan findings, palmoplantar pustulosis, and acne, a diagnosis of SAPHO syndrome was made. The patient was then seen by a rheumatologist and was started on 40 mg of daily prednisone and later transitioned to adalimumab 40 mg every 14 days. She was started on adjuvant Arimidex for her breast cancer and continues to follow with both oncology and rheumatology.

## 3. Discussion

SAPHO syndrome can present at any age but is most commonly found in children and young adults, and it has a self-limiting, relapsing, and remitting course [6, 7]. Diagnostic criteria for SAPHO syndrome include a combination of acne, palmoplantar pustulosis, hyperostosis with or without dermatosis, and recurrent multifocal chronic osteomyelitis involving the axial or peripheral skeleton, with or without dermatosis [5]. Synovitis of the affected joint is also a common presentation of SAPHO syndrome. Patients can also present with mono or polyarthritis [8]. Various skeletal sites are found to be affected in SAPHO syndrome, including anterior chest wall (65% to 90% of cases), spine (30% of cases with thoracic spine being the most frequently affected part of spine), appendicular skeleton including long bones (5% to 10% of cases), and mandibular area (1% to 10% of cases) [6, 7, 9]. Dermatological manifestations include palmoplantar pustulosis and severe acne [8]. Osteoarticular and dermatological manifestations do not necessarily occur at the same time as the latency period of more than ten years has also been documented [10]. Lab findings generally include moderate leukocytosis, mild anemia, elevated acute phase reactants, elevated immunoglobulin A, and normal or elevated complement levels [11].

Pathogenesis of SAPHO syndrome remains unknown. Multiple mechanisms, including genetic, immunological, and bacterial causes, are found to play an important role in the development of the disease. Regarding infectious etiology, *Propionibacterium* acnes have been isolated in biopsy specimens of both the bone and synovium [7, 8], but despite this, antibiotic trials are not found to be effective [7]. Three hypotheses have been proposed by Hellmann and Schwartz regarding autoimmune response playing an important role in pathogenesis of SAPHO syndrome, which could be triggered by a bacterial or viral pathogen [12, 13]. According to molecular mimicry hypothesis, whenever there is a resemblance between the fragment of microorganism in the skin with the bone or joint, the immune system mistakenly attacks the normal bone or joint causing inflammation of affected joints [14]. According to the second hypothesis, whenever a fragment of microorganism couples with an immunoglobulin, an immune complex is formed which deposits in the bone or joint and can activate a complement cascade, thus causing inflammation [15]. Finally, according to the third hypothesis, which is an immune barrier breakdown hypothesis, whenever a skin infection breaks down a barrier between immune cells and dermatologic tissues, normal antigens in the skin are exposed to the immune system, which may trigger an inflammatory reaction [16]. In some studies, association with seronegative spondyloarthropathies has also been found [7]. HLA B27 has also been found in 30% of SAPHO patients [8]. The main consideration as to why a spondyloarthropathy was not likely in our patient was the absence of several key elements including an antecedent infection, sacroilitis, or ossification of intervertebral ligaments on imaging, no history of inflammatory bowel disease, psoriatic rash or ocular symptoms.

The relationship between cancer and rheumatologic conditions is bidirectional, in that some conditions predispose one to cancer and some cancers may present with rheumatologic features. There is a known association between rheumatoid arthritis, lupus, Sjögren's syndrome, systemic sclerosis, and hematologic malignancies such as lymphoma and leukemia [17]. Furthermore, the presence of dermatomyositis/polymyositis may be a clue for an occult neoplasm [18]. Conversely, paraneoplastic rheumatic syndromes can develop as a result of an underlying malignancy. Hypertrophic osteoarthropathy (HOA) is one such process and has a well-known association with lung cancer. HOA is caused by abnormal growth of bone and skin in the distal extremities and classically presents as digital clubbing, symmetric polyarthritis, and periostitis of long bones [19]. The etiology is unclear, but believed to be caused by tumor-derived growth factors such as platelet-derived growth factor (PDGF) and vascular endothelial growth factor (VEGF) [20]. Typically, signs and symptoms resolve after treatment of the underlying disease. The absence of clubbing, tenderness of long bones, and lack of periosteal reaction on radiographs made HOA in our patient highly unlikely. Carcinomatous polyarthritis (CP) is associated with various solid tumors and can present similar to seronegative rheumatoid arthritis with symmetric involvement of hands, wrists with morning stiffness, or as an asymmetric migratory polyarthritis [21]. The classic presentation, however, is advanced aging, acute onset of symptoms, involvement of lower extremities, and lack of destructive changes on radiographs [22]. Other types of paraneoplastic rheumatic conditions include remitting seronegative symmetrical synovitis with pitting edema which is thought to be related to increased levels of VEGF [23], palmar fibromatosis, and polyarthritis which causes flexion deformities of the hands in patients with certain solid tumors [24]. Our patient's history and symptomatology was not consistent with any of the paraneoplastic rheumatoid conditions.

For diagnosis of SAPHO syndrome, whole-body scintigraphy is useful because in addition to increased tracer uptake in the affected bone, it also reveals clinically silent lesions. The bull's head sign is the pathognomonic of SAPHO syndrome which is characterized by manubrium sterni which represents the upper skull and the inflamed sternoclavicular joint with the adjacent clavicle representing the horns [25]. Though this sign is pathognomonic for SAPHO syndrome, it is not sensitive [26]. Plain radiographs, CT scans, and MRI are also found to be helpful in diagnosing SAPHO syndrome. Histological characteristics of bone lesions involve acute inflammation with predominantly a neutrophilic infiltrate [8]. The differential diagnoses of SAPHO syndrome include osteosarcoma, infectious osteomyelitis, bony metastasis, Ewing's sarcoma, Paget's disease, sternoclavicular osteoarthritis, infectious spondylodiscitis, and many others [8].

Regarding treatment of SAPHO syndrome, nonsteroidal anti-inflammatory drugs (NSAIDs) are the first line of agents [6]. But a lot of patients do not get any relief from NSAIDs alone. *Propionibacterium* acne, which is found to play an important role in pathogenesis of SAPHO syndrome, can be treated with antibiotics that have been found to decrease the disease activity in one of the studies [27]. Corticosteroids, colchicine, and disease-modifying antirheumatic drugs (DMARDs), including methotrexate and sulfasalazine, are also found to be effective in some of the case studies [8]. In patients who are refractory to the abovementioned treatment, biological agents such as tumor necrosis factor inhibitors including infliximab, etanercept, or adalimumab have also been used with varying results [6]. Bisphosphonates which inhibit bone resorption and also have probable anti-inflammatory properties have also been used in patients who are refractory to the NSAIDs, corticosteroids, colchicine, and DMARDs [6]. Finally, surgical procedures including partial or complete resection, saucerization, and decortication of the affected bone have also been shown to be useful in some of the cases [6].

## 4. Conclusion

The course of the disease is highly variable, owing to the fact that not all signs may be present at the same time. Recent studies show that 13% of patients had monophasic disease, and 35% of the patients had relapsing and remitting course [28]. Predictors of chronic disease include female gender, involvement of anterior chest wall, dermatologic lesions, peripheral arthritis, and elevated levels of acute phase reactants [6]. SAPHO syndrome is a distinct clinical entity with characteristic dermatologic and osteoarticular abnormalities and should be considered in the differential diagnosis of sclerotic and hyperostotic bone lesions in low-stage cancers to prevent the use of chemotherapy and radiotherapy in patients with malignancy [8]. Management of a patient with SAPHO syndrome is multidisciplinary in nature [1, 29], and a high index of suspicion is required to diagnose and treat this condition.
